# Intravenous Lidocaine: Old-School Drug, New Purpose—Reduction of Intractable Pain in Patients with Chemotherapy Induced Peripheral Neuropathy

**DOI:** 10.1155/2017/8053474

**Published:** 2017-03-28

**Authors:** Sandra A. S. van den Heuvel, Selina E. I. van der Wal, Lotte A. Smedes, Sandra A. Radema, Nens van Alfen, Kris C. P. Vissers, Monique A. H. Steegers

**Affiliations:** ^1^Department of Anesthesiology, Pain and Palliative Medicine, Radboud University Nijmegen Medical Center (RUNMC), Nijmegen, Netherlands; ^2^Department of Medical Oncology, RUNMC, Nijmegen, Netherlands; ^3^Department of Neurology, Clinical Neurophysiology, Donders Center for Neuroscience, RUNMC, Nijmegen, Netherlands

## Abstract

*Background.* Treatment of intractable pain due to chemotherapy induced peripheral neuropathy (CIPN) is a challenge. Intravenous (iv) lidocaine has shown to be a treatment option for neuropathic pain of different etiologies.* Methods.* Lidocaine (1.5 mg/kg in 10 minutes followed by 1.5 mg/kg/h over 5 hours) was administered in nine patients with CIPN, and analgesic effect was evaluated during infusion and after discharge. The immediate effect of lidocaine on pressure pain thresholds (PPT) and the extent of the stocking and glove distribution of sensory abnormalities (cold and pinprick) were assessed.* Results.* Lidocaine had a significant direct analgesic effect in 8 out of 9 patients (*P* = 0.01) with a pain intensity difference of >30%. Pain reduction persisted in 5 patients for an average of 23 days. Lidocaine did not influence mean PPT, but there was a tendency that the extent of sensory abnormalities decreased after lidocaine.* Conclusion.* Iv lidocaine has direct analgesic effect in CIPN with a moderate long-term effect and seems to influence the area of cold and pinprick perception. Additional research is needed, using a control group and larger sample sizes to confirm these results.

## 1. Introduction

Chemotherapy induced peripheral neuropathy (CIPN) is a clinically relevant side effect of various chemotherapeutic drugs that prevents an optimal treatment regimen in a significant proportion of patients [1, 2]. Although CIPN has been described with many chemotherapeutic drugs, the occurrence and severity of CIPN depend on the type of chemotherapeutic drug, regimen, cumulative dose, and individual patient factors [3]. CIPN has a relatively high prevalence, varying from 68.1% within the first month after cessation of chemotherapy to 30% at six months or later [4]. Several mechanisms have been described to explain the underlying pathophysiology of CIPN. Alterations in ion channel function, mainly sodium channels, are believed to play an important role in the pathophysiology of CIPN. These alterations induce changes in the cell membrane leading to spontaneous electric discharges, resulting in a hyperexcitability state of the nervous system [5, 6]. Additionally, increased release of inflammatory cytokines is believed to contribute to occurrence of chronic neuropathic pain and CIPN [3, 7].

Clinically, CIPN mainly manifests as a sensory peripheral neuropathy, although motor and autonomic nerve fibers may also be involved [2, 4]. The sensory neuropathy is often distributed in a “stocking and glove” manner (i.e., affecting the feet, distal lower legs, and hands) and causes symptoms like pain, sensory loss, allodynia, paresthesia, numbness, and tingling [2, 3, 8]. Symptoms frequently affect patients' daily activities and decrease their quality of life [2, 3]. Until now, no sufficient treatment option has been available for CIPN. Intravenous (iv) lidocaine has shown to be a successful option in treating various causes of chronic neuropathic pain [5, 9–11]. The first description of iv lidocaines' analgesic effect was in 1961, but with a high incidence of side effects at doses required for pain relief [12]. A report of Boas et al. in 1982 led to the analgesic use of lidocaine incline [13]. Lidocaine, a nonspecific sodium channel blocker, reduces ectopic nerve discharges, relieves hyperalgesia, and modulates the inflammatory response, because of an inhibitory effect on sodium, calcium, and potassium ion channels, G-protein coupled pathways, NMDA receptors, and the glycinergic system [14]. No study has been conducted yet to determine the effect of iv lidocaine on neuropathic pain in patients with CIPN, but theoretically it might be a potentially useful treatment modality. We experienced that iv lidocaine had an analgesic effect in individual patients with CIPN, and in some of them the stocking and glove distribution of sensory deficits changed. Therefore, our primary aim was to investigate the effect of a single infusion of iv lidocaine on neuropathic pain in patients with CIPN, and additionally we examined sensory symptoms.

## 2. Material and Methods

### 2.1. Patients

Patients were recruited in the period of January 2015 until November 2015 from the outpatient pain clinic of the Radboud University Medical Center, Nijmegen, The Netherlands. Patients aged 18 years or older, diagnosed with CIPN, who had a pain score of 5 or more on an 11-point numeric rating scale (NRS), were included. Exclusion criteria were last chemotherapy infusion < 12 weeks ago, myocardial ischemia < 6 months ago, cardiac arrhythmias or use of antiarrhythmic medication, nephropathy (glomerular filtration rate < 60 ml/min/1.73 m^2^), liver disease (serum bilirubin > 1.5 × above normal), hypokalemia, a known allergy for local anesthetics of the amide-type, diabetes mellitus or other known peripheral neuropathic disease, and pregnancy or lactation. Concomitant use of other analgesics drugs at the moment of inclusion was continued if necessary, without dosage modifications. Demographic characteristics like age, gender, medical history, previous analgesics usage, and Hospital Anxiety and Depression Scale (HADS) were collected. Patients completed the National Cancer Institute-Common Toxicity Criteria (NCI-CTC) questionnaire before treatment with lidocaine. The NCI-CTC questionnaire is used to determine the grade of neuropathy, ranging from 0 to 4, where 0 corresponds to no neuropathic symptoms and 4 to the most severe neuropathic symptoms [15]. The HADS is a questionnaire to determine the level of anxiety and depression, with a maximum of 42 points [16, 17]. A total HADS > 12 (sensitivity 0.80 and specificity 0.74) indicates a state of depression or emotional distress [18]. The study protocol was approved by the institutional review board and participants gave informed consent to use their data.

### 2.2. Treatment

The study used a prospective observational cohort design. Patients received the following regimen of iv lidocaine: a bolus of 1.5 mg/kg, infused in 10 minutes, followed by continuous infusion of lidocaine 1.5 mg/kg/h over a 5-hour period. This infusion algorithm is selected since the analgesic effect seems to be correlated with duration of infusion [11], and therapeutic plasma levels for pain treatment are within 1–5 *μ*g/ml [19, 20]. Plasma levels of 2–4 *μ*g/ml will be achieved with a bolus of 2 mg/kg followed by continuous infusion 2-3 mg/kg [21], though awake patients can experience unwanted side effects when using these dosage schemes.

A three-lead electrocardiogram, blood pressure, and oxygen saturation were monitored continuously throughout the study by an anaesthesiologist. Patients were observed for possible side effects till approximately 30–60 minutes after treatment with lidocaine; during this period no study measurements were performed. All measurements were performed by two clinicians (physician and medical student).

### 2.3. Clinical Pain Score

The intensity of pain was assessed using the numeric rating scale (NRS, 0: no pain, 10: worst pain imaginable) [22]. Pain ratings were obtained directly before start of infusion, every 15 minutes during the first hour, and subsequently every 30 minutes until the end of infusion. During lidocaine infusion, pain ratings were obtained separately for both hands and both feet. Correlation between duration of infusion and pain ratings was assessed. The difference between the NRS at baseline and the NRS after treatment with iv lidocaine was used to calculate the absolute pain intensity difference (PID) and the percentage pain intensity difference (PID%). An absolute decrease in pain intensity scores of ≥2 points on the NRS or a PID% of ≥30% was considered clinically significant [23]. Patients with a clinical significant result were defined as responders. The other patients were seen as nonresponders.

The duration of the analgesic effect after discontinuation of lidocaine infusion was assessed by number of consecutive days till pain was returned to baseline. Patients received a pain diary, in which pain scores were asked 3 times daily, from days 0–10 every day, from day 14, and further weekly. They were called by the physician every 3 weeks after infusion, who asked if there has been any prolonged analgesic effect and what the duration of this effect was. If pain was returned to baseline, alternative pain management was discussed; and if there was still pain reduction, they were called again after 3 weeks.

### 2.4. Examination Sensory Symptoms

Pain sensitivity and sensory changes of the hand and feet were examined directly before and after treatment with lidocaine. The order of examination was successively as follows: (1) pressure pain thresholds, (2) mechanical sensory testing, and (3) thermal sensory testing.

#### 2.4.1. Pressure Pain Thresholds

Baseline pain sensitivity was assessed by measuring the responses evoked by mechanical non invasive stimuli, that is, pressure pain thresholds (PPT) [24]. PPT assessment has proven to be a method with satisfactory intraindividual reproducibility and reliability [25, 26] and can easily be determined by a trained professional with a pressure algometer [27].

A hand-held pressure algometer (Wagner force ten™ FDX digital force gage) with a contact area of 1 cm^2^ was used to give a standardized pressure stimulus of 50 kPa/s. PPT was assessed on both the right and left body side at the following locations: m. trapezius pars medialis, thenar eminence, m. rectus femoris (15 cm above patella), and the m. abductor hallucis. Patients received instructions to notify the investigator if their PPT was reached; subsequently the associated pain score (NRS) was obtained. To familiarize patients with the method of testing, measurements started with a pressure stimulus on the ventral side of the chest.

#### 2.4.2. Mechanical and Thermal Sensory Testing

**Figure 1:**
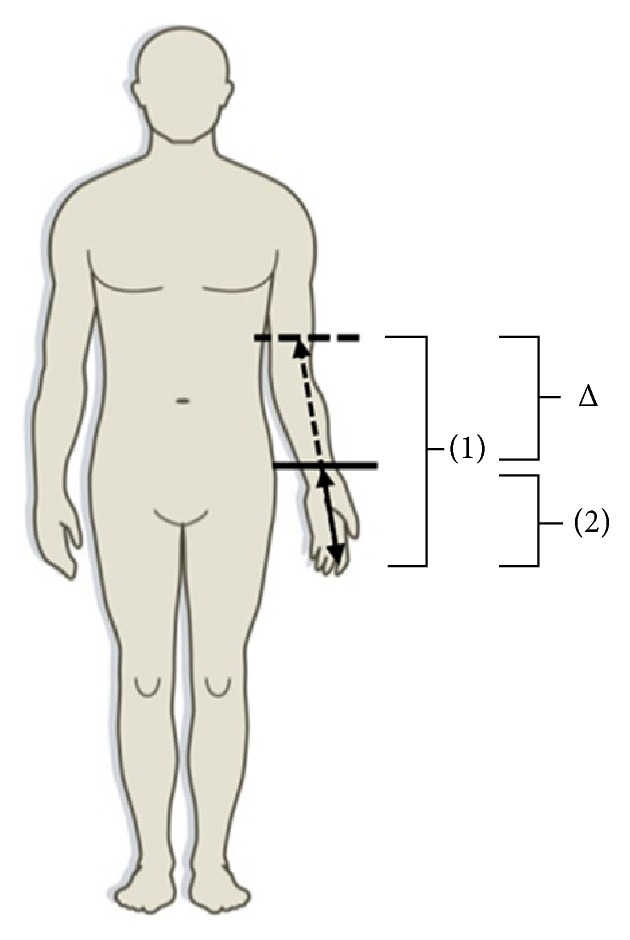
Method of thermal and mechanical testing. (1) Distance between point of sensory change and digitus III before iv lidocaine. (2) Distance between point of sensory change and digitus III after iv lidocaine. Δ: change in distance before and after treatment with iv lidocaine.

The distribution of the sensory neuropathy was measured with a Tip-Therm stored on ice and a MRC PinPrick stimulator of 256 mN. In order to assess the location of sensory changes, cold and pinprick stimuli were repeatedly delivered on the arm and leg. Stimuli were given from the anterior attachment of the m. deltoideus to digitus III, respectively, from the top of the patella to the hallux. Patients had to indicate the location of sensory change. The distance (cm) between this position and, respectively, digitus III or the hallux represented the extent of the distribution (Δ). The method of testing is shown in Figure 1. To familiarize patients with the method of testing, measurements started with a pinprick and cold stimulus on the ventral side of the chest.

### 2.5. Statistical Analysis

Continuous data were expressed as means ± standard deviation or as means with a two-tailed 95% confidence interval in case of normally distributed data. The Kolmogorov-Smirnov test was used to assess if data were normally distributed. Skewed data are presented as median with interquartile range (IQR). Paired data were analyzed using the paired *t*-test or the Wilcoxon signed rank test, depending on the distribution. A Pearson correlation coefficient was calculated to detect a correlation between duration of infusion and NRS. The Bonferroni correction was used for multiple comparisons adjustment. Statistical Package for Social Sciences, Version 22.0 (IBM Corporation, Armonk, NY, USA), was used for statistical analysis. Figures were created using GraphPad Prism, Version 5.03 (GraphPad Software Inc., La Jolla, CA, USA). A *P* value less than 0.05 was considered to be statistically significant. The Bonferroni corrected *P* value considered for statistical significance is equal to 0.006.

## 3. Results

### 3.1. Patient Characteristics

Nine patients, 4 males and 5 females (mean age 52.7 ± 11.9), were included during the study period. Patient characteristics are depicted in Table 1. Patients were treated for various types of malignancies with one or more chemotherapeutic agents, which all could induce CIPN. Seven patients suffered neuropathy grade 4; 1 patient reported neuropathy grade 2; and another reported neuropathy grade 1 on the NCI-CTC scale. The median of the HADS value was 17 (IQR: 8.25–18.25). All patients reported pain in the lower extremities; 6 patients also experienced pain in the upper extremities. Most patients had already tried a wide variety of analgesics (*e.g.,* NSAIDS, opioids, antidepressants, and antiepileptics), but none were previously treated with iv lidocaine. During treatment with lidocaine no serious side effects occurred. All patients remained respiratory and hemodynamically stable (no cardiac arrhythmias were observed) and could be discharged within one hour after infusion.

### 3.2. Effect on Clinical Pain Score

Treatment with iv lidocaine caused a clinically significant decrease in the NRS score at group level (*P* = 0.01). Eight out of 9 patients experienced an analgesic effect of lidocaine. The mean NRS score was 7.7 (NRS 5–9) before infusion and dropped to a mean NRS score of 3.1 (NRS 0–7) after treatment.

**Figure 2:**
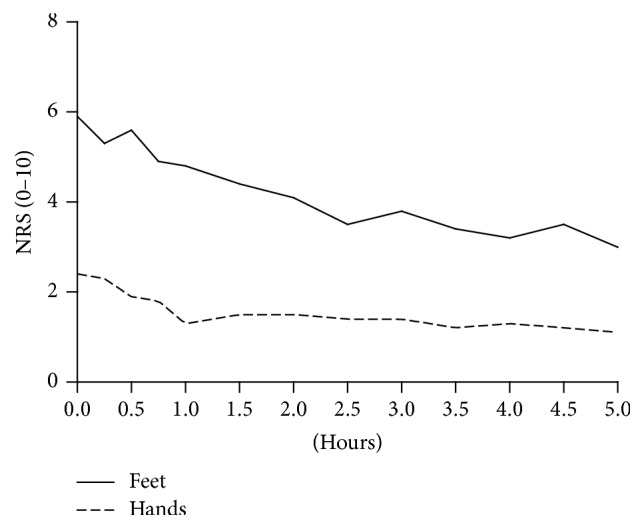
Comparison of NRS scores of hands and feet during lidocaine infusion. Data are expressed as means.

The effect of iv lidocaine on pain (NRS scores) for every patient is presented in Table 2 and the change in mean NRS scores during infusion is displayed in Figure 2. NRS scores of both hands and feet correlated significantly with duration of infusion; the correlation coefficient of hands and feet was −0.8 (*P* < 0.01) and −0.9 (*P* < 0.01), respectively. At the start of infusion patients reported a higher NRS score for their feet (5.9 ± 2.2) compared with their hands (2.4 ± 2.3). Subsequently, the NRS of the feet showed a larger decrease than the NRS of the hands.

In three patients experiencing pain reduction during lidocaine treatment, the analgesic effect disappeared almost immediately after discontinuation of the infusion. Five patients experienced a more sustained analgesic effect, which varied from 3 days up to 56 days with a mean duration of 23 days.

### 3.3. Pressure Pain Thresholds

Comparable results were obtained when PPTs and associated NRS scores were expressed separately for the left and right body side; therefore the means of left and right side measurements were used for analysis. The effect of iv lidocaine on the pressure pain thresholds (PPT) and associated NRS scores is shown in Table 3. There was no significant effect of iv lidocaine on the mean PPT and associated NRS score. The effect on PPT before and after iv lidocaine varied between −2.31 and 1.16 N/cm^2^ (*P* = 1); and for associated NRS score the effect varied between −0.67 and 0.06 NRS points (*P* = 0.204–1).

### 3.4. Thermal and Mechanical Sensory Testing

Although individual patients could have an asymmetrical distribution of their stocking and glove distribution, no statistically significant difference was found between left and right side for the distribution of sensory changes for pinprick and cold in the whole group. Combining both limbs before and after lidocaine infusion, the cold detection level was significantly more caudal as the pinprick detection level (*P* = 0.008; difference = 6.1 cm; 95% CI [1.7–10.6]) was found. For limbs and infusion separately, the pinprick detection level was always more cranial as the cold detection level; however, in these subgroups with less observations the difference is not always statistically significant (*P* varies between 0.018 and 0.625). In 1 patient no changes in cold or pinprick sensation were observed.

#### 3.4.1. Influence of Lidocaine on Thermal and Mechanical Sensory Testing

The changes in distribution of pinprick and cold sensation in individual patients are presented in Table 4.

In 4 patients the area of abnormality in cold perception decreased after infusion. Three patients reported an increase in area in the upper or lower extremity after infusion. One patient showed a combination of positive change in one leg and a negative change in the other leg. The area of cold perception decreased when combining the limbs in all patients (*P* = 0.292; difference = 3.7 cm; 95% CI [−3.5–10.8]).

In 4 patients the area of abnormal pinprick sensation in the upper or lower extremities decreased after infusion; in 1 patient it increased in both upper and lower extremities. Three patients showed a combination of both increase in one area and decrease in another. The area of abnormal pinprick sensation decreased when combining the limbs in all patients (*P* = 0.099; difference = 6.4 cm; 95% CI [−1.3–14.1]).

## 4. Discussion

A single infusion of lidocaine decreased pain in 8 out of 9 patients with CIPN, which was correlated with duration of iv lidocaine infusion. The long-term analgesic effect of lidocaine was moderate with a mean duration of 23 days. Lidocaine had no effect on pain sensitivity measured by PPT. The distribution of sensory abnormalities was influenced by lidocaine.

There have been other studies that assessed the effect of iv lidocaine on neuropathic pain, but to our knowledge no study has specifically investigated the effect of lidocaine in CIPN. A Cochrane review that included 30 RCTs showed that iv lidocaine and its oral analogues, mexiletine and tocainide, reduce neuropathic pain [9]. This review included heterogeneous studies that looked at various etiologies of peripheral neuropathic pain (e.g., diabetic, posttraumatic, and central pain) and lidocaine dosages also varied (1–5 mg/kg in 30–60 minutes) between studies. Tremont-Lukats et al. investigated three dosing regimens of lidocaine (1, 3, and 5 mg/kg/hr during 6 hours) in various neuropathic pain syndromes. Pain scores continued to decrease till 4 hours after start of lidocaine infusion. Lidocaine at 5 mg/kg/hr was more effective at relieving neuropathic pain than lower dosages, and this effect persisted for at least 4 hours after end of infusion [11]. Three other studies investigated the effect of lidocaine in homogenous patient groups. In two small groups of patients with peripheral nerve injury, lidocaine (5 mg/kg in 30 minutes or 4 hours) had conflicting analgesic efficacy and long-term effects were not evaluated [28, 29]. Lidocaine (5 mg/kg or 7.5 mg/kg in 4 hours) had a significant effect in reducing pain in a group of 15 patients with intractable diabetic neuropathy. This effect lasted for up to 28 days [30], which is comparable to our results.

### 4.1. Sensory Testing

PPT is increasingly used to compare pain sensitivity before and after treatment [31, 32]. No influence of lidocaine was found on PPT and associated NRS. Previous studies investigating the analgesic effect of agents targeting neuronal excitability have shown that pretreatment pressure pain thresholds can predict analgesic efficacy of treatment [33]. In our study, no correlation between pressure pain thresholds and efficacy of treatment was found. The mean pressure pain thresholds in our population were comparable to thresholds described in healthy individuals [34].

Spread of sensory abnormalities showed a marked variance between patients; however the mean affected area for cold detection had a tendency to be smaller than for pinprick. Cooling the skin to 4°C activates A-*δ* and C-fibers sensitive to innocuous cooling and cold-sensitive nociceptors. The perception of pinprick pain intensity is related to activity in A-*δ* fiber nociceptors [35]. Krøigård et al. observed in patients with CIPN caused by oxaliplatin and docetaxel that mechanical detection threshold measured with von Frey hairs was more affected as the cold detection threshold [36]. These results indicate that CIPN affects both small and large nerve fibers. It has to be noted that some patients found it challenging to indicate the exact location of sensory change, possibly resulting in a test with a lower accuracy and sensitivity.

The effect of lidocaine on the distribution of sensory abnormalities showed unexpected results, with an increase of sensory abnormalities in some and a decrease in other patients. Although these changes were not statistically significant, it is interesting that a pharmacological agent can influence and maybe decrease sensory abnormalities. These results could provide a basis for further research on developing treatments, including specific sodium channel blockers, which reduce or even treat sensory disturbances in polyneuropathy.

CIPN is known to be a common dose limiting side effect of chemotherapeutic agents like taxanes, vinca alkaloids, and platinum compounds [1, 2]. Proposed mechanisms for taxane-induced neuropathy are a disrupted axonal microtubule structure and a toxic effect on mitochondria in primary afferent neurons. Vinca alkaloids induce alterations in neuronal cytoskeleton leading to impaired axonal transport and degeneration. Platinum compounds accumulate in the dorsal root ganglia resulting in decreased cellular metabolism and axoplasmic transport [1–3]. Most of these agents will be combined with other types of chemotherapy in cancer treatment, like antimetabolites, topoisomerase inhibitors, or antitumor antibiotics, which can have equally a neuropathy as a side effect.

In this study, patients with various types of malignancies were included, who were also treated with different chemotherapeutic agents, which may have resulted in heterogeneity of their sensory profiles. From these data no conclusions can be drawn which sensory abnormalities can be seen in CIPN caused by a specific type of chemotherapy or which responds best to lidocaine treatment. Since several trials indicate that sensory phenotyping can predict drug responsiveness [37, 38], a larger and more detailed study could untangle the different pathophenotypes of CIPN and their responsiveness to treatment. Drawbacks of our study are its small sample size and lack of control group. However, despite the small sample size, a significant direct analgesic effect was observed, even though patients received various types of chemotherapy and had different comorbidities. Additionally, most patients had severe neuropathy and had previously received various analgesics and the median HADS value was 17 [39]. These factors can render a subject more susceptible to nonsuccessful pain treatment.

## 5. Conclusion

Iv lidocaine, an old-school drug, significantly reduced intractable pain in patients with CIPN for an average of 23 days in our study and there was a tendency for a decreased extent of sensory abnormalities. Our results are therefore promising and show a potential role for iv lidocaine in patients with CIPN, when standard treatment algorithm for neuropathic pain fails [40]. This might prevent them from finishing the intended optimal chemotherapy regimen. Furthermore, patients who do not respond with a long lasting effect to lidocaine can be prescribed an oral sodium channel blocker like mexiletine, carbamazepine, or lamotrigine. These promising results provide a basis for the development of larger randomized trials, investigating the role of lidocaine in the treatment of CIPN.

## Figures and Tables

**Table 1:** Clinical characteristics.

Patient number	Sex/age (yr)	Type of malignancy	Type of chemotherapy	NCI-CTC grade	Pain duration (mo)	HADS^*∗*^	Pain location	Analgesic treatment before study
1	M/62	Colorectal cancer	Oxaliplatin, capecitabine	4	10	19	Upper & lower extremity	Amitriptyline, pregabalin carbamazepine

2	M/45	Testis Carcinoma	Cisplatinum	4	22	6	Lower extremity	Amitriptyline, pregabalin nortriptyline, topiramate methadone, etoricoxib oxynorm

3	F/70	Colorectal cancer	Oxaliplatin	4	60	18	Upper & lower extremity	Amitriptyline, pregabalin duloxetine, diazepam acetaminophen

4	F/54	Breast Cancer	Docetaxel, capecitabine, cyclophosphamide, trastuzumab	4	16	17	Lower extremity	Amitriptyline, pregabalin carbamazepine, fentanyl oxynorm, diclofenac hydromorphone, acetaminophen

5	F/53	Chronic myeloid leukemia	Cyclophosphamide, imatinib	4	60	9	Upper & lower extremity	Amitriptyline, pregabalin tramadol, gabapentin ibuprofen, mirtazapine acetaminophen

6	M/53	Chronic lymphoid leukemia	Cisplatinum, cyclophosphamide, fludarabine, cytarabine, etoposide	4	30	—	Upper & lower extremity	Amitriptyline, pregabalin fentanyl, acetaminophen

7	F/32	Ovarian Cancer	Cisplatinum, paclitaxel, carboplatin	1	16	17	Lower extremity	Pregabalin

8	M/64	Non-Hodgkin Lymphoma	Cyclophosphamide, etoposide, cytarabine	4	180	—	Upper & lower extremity	Duloxetine, fentanyl oxynorm

9	F/41	Colorectal cancer	Oxaliplatin, capecitabine, bevacizumab	2	27	—	Upper & lower extremity	Oxycontin, oxynorm

M: male; F: female; NCI-CTC: National Cancer Institute-Common Toxicity Criteria; HADS: Hospital Anxiety and Depression scale. ^*∗*^HADS questionnaire is missing in 3 patients.

**Table 2:** Individual NRS scores before and directly after treatment and duration of analgesic effect of iv lidocaine.

Patient number	NRS before	NRS after	PID	PID%	Duration (days)
1	9	5	4	44	7
2	8	3	5	63	0
3	9	2	7	78	28
4	8	0	8	100	0
5	5	0	5	100	3
6	8	4	4	50	0
7	8	4	4	50	21
8	7	3	4	43	56
9	7	7	0	0	0

PID: pain intensity difference.

PID%: percentage pain intensity difference.

**Table 3:** PPTs and associated NRS scores before and after treatment with iv lidocaine.

	Location	Before^*∗*^	After^*∗*^	Effect	*T*-statistic	95% CI	*P* value^*∗∗*^
PPT	M. trapezius pars medialis	51.7 ± 14.3	52.0 ± 12.5	0.34	−0.086	[−9.6; 8.9]	1
	Thenar eminence	58.0 ± 18.2	58.3 ± 14.9	0.33	−0.070	[−11.4; 10.7]	1
	M. rectus femoris	71.3 ± 26.4	69.0 ± 16.2	−2.31	0.257	[−18.5; 23.1]	1
	M. abductor hallucis	55.6 ± 27.7	56.8 ± 18.3	1.16	−0.192	[−15.0; 12.7]	1

NRS	M. trapezius pars medialis	5.6 ± 2.0	5.4 ± 2.4	−0.11	0.187	[−1.3; 1.5]	1
	Thenar eminence	5.6 ± 1.9	5.1 ± 1.8	−0.56	2.294	[−0.0; 1.1]	0.204
	M. rectus femoris	4.9 ± 2.3	5.0 ± 2.0	0.06	−0.127	[−1.1; 1.0]	1
	M. abductor hallucis	6.3 ± 2.0	5.7 ± 2.0	−0.67	1.193	[−0.6; 2.0]	1

PPT: pressure pain threshold, N/cm^2^; NRS: numeric rating scale, scale 0–10.

^*∗*^Data are expressed as means ± SD; ^*∗∗*^Bonferroni-corrected *P* values are reported.

**Table 4:** The change in distance (Δ) for cold and pinprick stimuli before and after treatment with iv lidocaine.

Patient number	Cold				Pinprick			
	Arm		Leg		Arm		Leg	
	Right	Left	Right	Left	Right	Left	Right	Left
	Δ (cm)	Δ (cm)	Δ (cm)	Δ (cm)	Δ (cm)	Δ (cm)	Δ (cm)	Δ (cm)
1	−22	−14	−45	−3	−28	−22	−30	−41
2	0	−26	−8	−2	0	+7	+23	+17
3	−13	−11	−7	−3	−25	−16	−10	−6
4	0	0	0	0	0	0	0	0
5	0	0	+24	+45	+2	−2	−2	−3
6	0	0	0	+12	−7	−11	+27	+5
7	0	0	−8	−8	0	0	−26	0
8	0	+19	+1	0	+19	+3	−6	−6
9	0	0	−2	+6	0	0	−5	−2
